# Intraoperative Ultrasound: Bridging the Gap between Laparoscopy and Surgical Precision during 3D Laparoscopic Partial Nephrectomies

**DOI:** 10.3390/diagnostics14090942

**Published:** 2024-04-30

**Authors:** Ionela Mihai, Horatiu Dura, Cosmin Adrian Teodoru, Samuel Bogdan Todor, Cristian Ichim, Nicolae Grigore, Cosmin Ioan Mohor, Alin Mihetiu, George Oprinca, Nicolae Bacalbasa, Denisa Tanasescu, Dan Georgian Bratu, Adrian Boicean, Bogdan Oros, Adrian Hasegan

**Affiliations:** 1 Faculty of Medicine, Lucian Blaga University of Sibiu, 550169 Sibiu, Romania; ionela.mihai@ulbsibiu.ro (I.M.); adrian.teodoru@ulbsibiu.ro (C.A.T.); samuelbogdant@gmail.com (S.B.T.); cristian.ichim@ulbsibiu.ro (C.I.); nicolae.grigore@ulbsibiu.ro (N.G.); cosmin.mohor@ulbsibiu.ro (C.I.M.); alin.mihetiu@ulbsibiu.ro (A.M.); georgecalin.oprinca@ulbsibiu.ro (G.O.); denisa.tanasescu@ulbsibiu.ro (D.T.); dan.bratu@ulbsibiu.ro (D.G.B.); adrian.boicean@ulbsibiu.ro (A.B.); adrian.hasegan@ulbsibiu.ro (A.H.); 2 Surgery Department, University of Medicine and Pharmacy “Carol Davila” Bucharest, 020021 Bucharest, Romania; nicolaebacalbasa@gmail.com; 3 County Clinical Emergency Hospital of Sibiu, 550245 Sibiu, Romania

**Keywords:** partial nephrectomy, intraoperative ultrasound, laparoscopy, renal tumor

## Abstract

The use of 3D laparoscopic partial nephrectomy has emerged as a cornerstone in the surgical arsenal for addressing renal tumors, particularly in managing challenging cases characterized by deeply seated tumors embedded within the renal parenchyma. In these intricate scenarios, the utilization of intraoperative ultrasound (IOUS) acquires paramount importance, serving as an indispensable tool for guiding and meticulously monitoring the surgical process in real time. To further explore the efficacy of IOUS-guided techniques, we conducted a retrospective study comparing outcomes in patients who underwent partial nephrectomy with IOUS guidance (*n* = 60) between 2020 and 2022 with a cohort from 2018 to 2019 without IOUS guidance (*n* = 25). Our comprehensive analysis encompassed various post-operative parameters, including the duration until food resumption, analgesia requirements, and length of the hospital stay. While these parameters exhibited comparable outcomes between the two groups, notable distinctions emerged in the intraoperative metrics. The IOUS-guided cohort demonstrated significantly reduced blood loss, a shorter median operative duration, and diminished ischemia time (*p* = 0.001). These compelling findings underscore the undeniable benefits of IOUS-guided techniques in not only facilitating the attainment of negative surgical margins but also in enhancing procedural safety and precision, thereby contributing to improved patient outcomes in the management of renal tumors.

## 1. Introduction

Over the past few decades, the landscape of urology has been reshaped by remarkable technological advancements, fundamentally altering the strategies employed for diagnosing and treating renal conditions [1]. A crucial aspect of this evolution is the adoption of laparoscopic techniques, which have been accompanied by a significant decrease in intraoperative complications and postoperative morbidity, as well as shorter hospital stays and superior aesthetic outcomes compared to the results for traditional open surgical approaches [2]. Among these laparoscopic interventions, partial nephrectomy has emerged as a favored therapeutic approach for localized renal tumors, providing good oncological results with the benefit of preserving renal function [3].

Renal tumor formations can be classified in multiple ways, through both imaging and histology, and the choice of the optimal therapeutic method depends on staging [4]. Historically, the first planned nephrectomy was documented as being performed over a century ago and was carried out using traditional methods [5]. In contemporary times, technology has witnessed substantial advancements, expanding the scope of pathologies warranting intervention. Notably, renal cancers have shown remarkable improvements in outcomes [6]. Although radical nephrectomy was once a general option and considered the gold standard, today this intervention is reserved for specific cases, in accordance with the degree of renal involvement, the decision of the multidisciplinary team, and the patient’s preferences [7,8]. Few surgeons still consider this intervention as a first-line option, yet this technology remains controversial [9].

The technological advancement brought about by the widespread adoption of laparoscopy has revolutionized surgery across almost all specialties. This transition has been embraced and has further evolved over time, driven by the daily challenges faced not only by urologists and general surgeons but also by professionals in other medical fields. A major challenge in laparoscopic partial nephrectomy is the precise identification and delineation of tumors, especially when they are endophytic, located within the renal sinus [10,11]. Thus, there has been a need for the introduction of an intraoperative imaging method that allows for clear visualization of tumor margins, vascularization, and contact with the renal pedicle [12]. In this regard, the innovation lies in the introduction of laparoscopic intraoperative ultrasound, a promising tool that combines the advantages of laparoscopy with the precision of ultrasound imaging [13].

Laparoscopic intraoperative ultrasound relies on the use of a laparoscopically introduced ultrasound transducer to obtain real-time images of the renal pedicle, tumor, and peritumoral vascularization [12,14]. This capability empowers the surgeon to observe tumors that are either not discernible on the renal surface or are positioned adjacent to vascular structures, such as the collecting system. This real-time guidance can contribute to reducing the risk of positive surgical margins and avoiding the excessive removal of healthy renal tissue [15].

Additionally, in cases of intrarenal tumors or those near the renal hilum, laparoscopic intraoperative ultrasound can provide essential information for resection planning, thus avoiding major complications such as vascular or urinary injuries [16]. However, like any medical technique, laparoscopic intraoperative ultrasound comes with challenges, such as the learning curve associated with interpreting ultrasound images or maneuvering the probe in a limited space [17,18]. In contrast to other patented imaging methods on the market, ultrasound remains highly advantageous. It is non-radiating for both patients and medical personnel, allows for repetitive use, and entails relatively low usage costs. Moreover, the acquisition of equipment is not excessively expensive, and modern ultrasound machines offer exceptional functionalities, with high image quality [19].

In the context of the rapid evolution of minimally invasive techniques and medical imaging, it is crucial to understand the potential and limitations of laparoscopic intraoperative ultrasound [20,21].

This paper aims to explore the advantages of intraoperative laparoscopic ultrasound, emphasizing its clinical significance in partial nephrectomy, with a detailed discussion of its advantages, disadvantages, and prospects in modern renal surgery.

## 2. Material and Methods

Over a period of 5 years at the Urology Department of Sibiu County Clinical Hospital, we evaluated 85 patients with renal tumors T1a and T1b, based on a retrospective study. Intraoperative ultrasound was employed in 60 patients assessed from 2020 to 2022, while the remaining 25 patients were evaluated from 2018 to 2019, without the benefit of intraoperative ultrasound due to a lack of technical resources. The surgical interventions were performed by the same surgical team for all patients. The inclusion criteria for patients were: adults with renal tumors T1a and T1b, with complete data, and with the entire course of treatment received at the aforementioned clinic. All patients underwent a standardized preoperative evaluation, including blood tests, abdominal ultrasound, native or contrast-enhanced CT examination and where necessary, abdominal and pelvic MRI. The goal of these investigations was to precisely identify the tumor’s location, exclude metastases, and assess the presence of any potential tumor thrombus in the renal or inferior vena cava. Preoperatively, all patients undergoing surgery received a central venous catheter and after general anesthesia induction with endotracheal intubation, a urethral-vesical catheter and a nasogastric tube were placed. An epidural catheter was mounted in all patients for improved postoperative analgesia.

For intraoperative ultrasound procedures, a Hitachi L44LA ARIETTA 60 diagnostic unit with a linear LUS-type IPX7 range probe was utilized, featuring a frequency of 7.0 MHz. The probe consists of an operating head containing the working handle, which can be adjusted for left or right deflection of the ultrasound area. The probe is equipped with a protective tube that can be inserted through the 12 mm trocar.

Laparoscopic intraoperative ultrasound was utilized in 60 out of the 85 patients to provide guidance. Among these procedures, 40 tumors were endophytic (completely incorporated into the renal parenchyma), while 20 were exophytic (protruding from the kidney surface).

During the surgery, once the tumor was identified and isolated, the LUS (laparoscopic ultrasound) probe was employed for exploration. The exploration procedure included determining the position of the renal tumor formation, along with its depth, size, and vascularization, as well as confirming the area and depth of excision. Following this, laparoscopic ultrasound (LUS) was employed to examine the artery and vein at the renal hilum. Following tumor resection and after ensuring hemostasis, the renal artery was declamped, and the laparoscopic probe was used to observe the restoration of blood flow in the operative area. The primary objective of this methodology was to preserve tumor margins and ensure complete resection, as well as to provide constant monitoring of the renal vascular status during the intervention.

**Figure 1:**
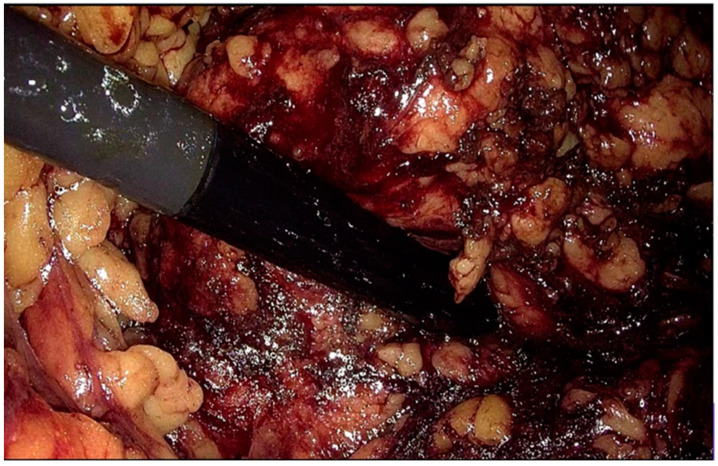
IOUS (intraoperative ultrasound) probe through the retroperitoneal space at the level of the kidney surface (personal collection).

**Figure 2:**
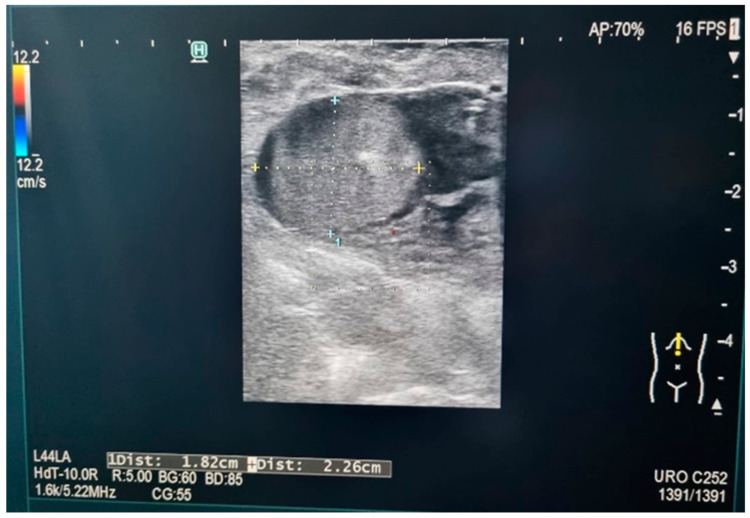
Tumor position and size (personal collection).

**Figure 3:**
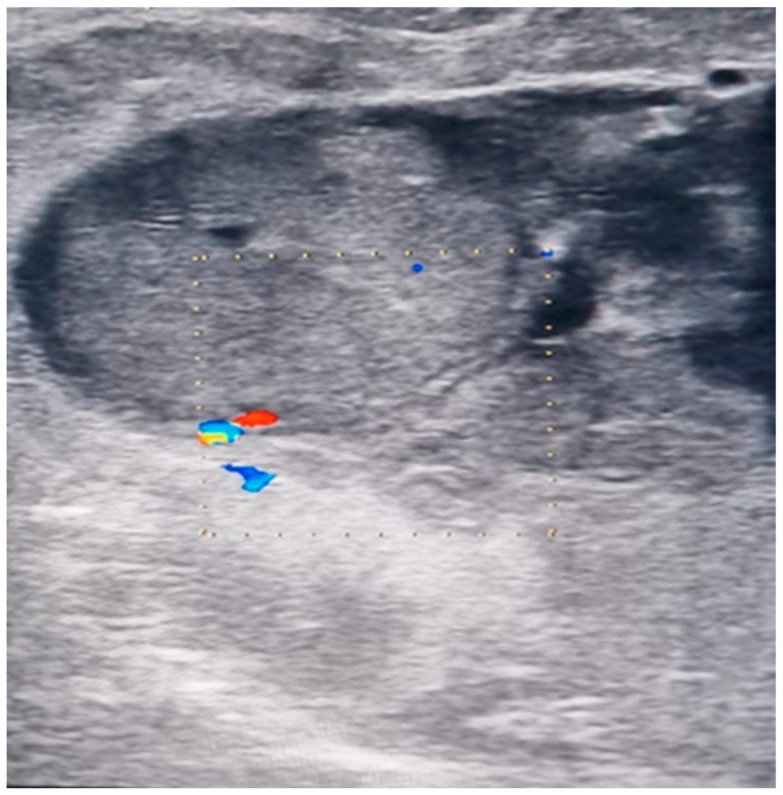
EcoDoppler image of the tumor and vascularization (personal collection).

**Figure 4:**
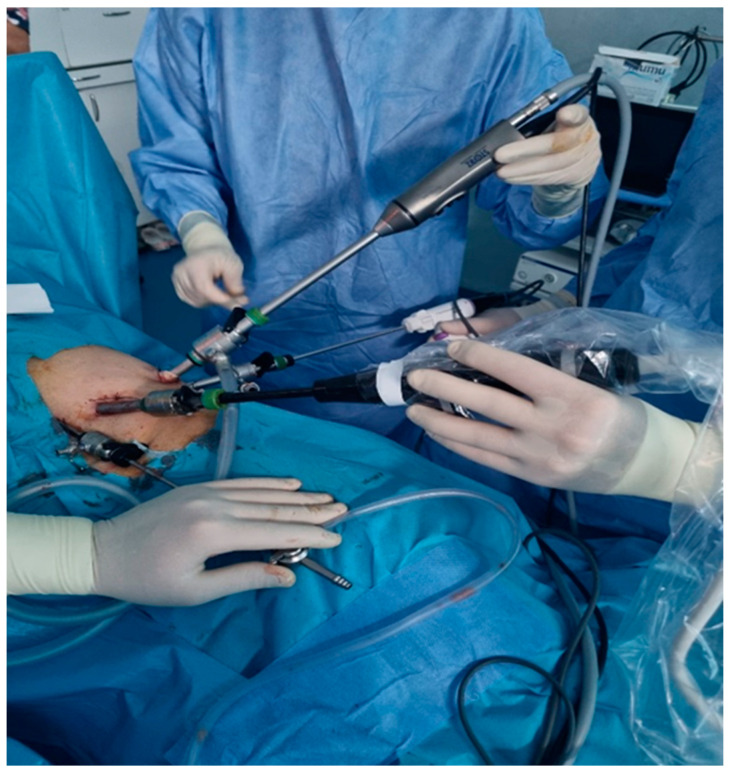
The position of the trocars and ultrasound probe inserted through the 12 mm trocar (personal collection).

**Figure 5:**
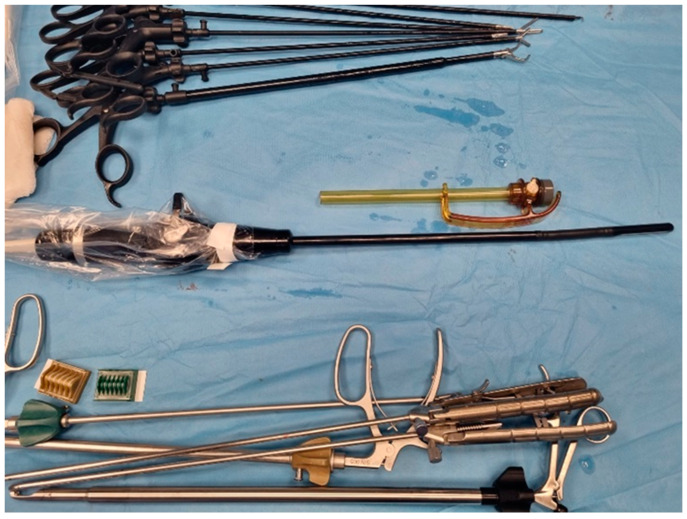
Laparoscopic instrumentation and LUS-type IPX7 range probe (personal collection).

The sterile ultrasound probe is inserted through the 12 mm trocar after being applied to the tip of the sterile Chatejel probe. Identification of the renal tumor margin is achieved by Doppler velocimetry by positioning the probe above the tumor and identifying the lesion and healthy renal tissue. After tumor identification, the distance to the renal sinus, tumor depth, and vascularization are recorded. Tumor circumferential dissection is performed using a hook clamp, and then the endocavitary ultrasound probe is withdrawn. The renal artery is clamped using a bulldog and in selected cases, selective clamping is used. The renal vein is clamped if the renal tumor is located in the renal hilum or near the renal vein, as well as in the case of right partial nephrectomy, in order to prevent reflux bleeding. The opening of the pyelocaliceal system and tumor excision is performed using cold scissors, the tumor vessels are clamped with Hem-o-lok clips, and are then resected, with macroscopic verification of tumor margins throughout excision. After resection, the tumor formation is placed directly into the endoscopic bag. Renorrhaphy is performed with 2.0 PDO’x Polydioxanone sutures in two layers through continuous suturing at the renal medulla level by closing the pyelocaliceal system, and a Hem-o-lok clip is placed at the ends of the suture. Then, continuous cortical renal suturing is performed with the placement of Hem-o-lok clips at the entrance and exit from the renal parenchyma of the suture, and closure of the suture is also achieved by clip placement. Renal artery and/or vein declamping is performed, and the endocavitary ultrasound probe can be used to visualize the restoration of blood flow in the resection area (Figure 1, Figure 2, Figure 3, Figure 4 and Figure 5).

Statistical analysis was performed using IBM-SPSS software version 22.0. Continuous data were presented as the median (IQR-interquartile range) and compared using a Mann–Whitney U test. Categorical variables were presented as count (percentages) and compared using Fisher’s exact test. The p-value was considered statistically significant if it was less than 0.05.

In this study, we conducted a power analysis to determine the likelihood of detecting significant effects based on our sample size of 85 patients. We used the general linear model to calculate power, considering an assumed effect size, a significance level (alpha) of 0.05, and a desired power level of 0.80. The achieved power was then reported to indicate the reliability of our findings.

## 3. Results

Our retrospective study examined 85 patients who underwent laparoscopic partial nephrectomy between 2018 and 2022. During the period from 2020 to 2022, laparoscopic intraoperative ultrasound (IOUS) was employed for a cohort of 60 patients, while 25 patients during the 2018–2019 period did not benefit from this technique. We analyzed the general characteristics of these patients, as presented in Table 1.

Among patients who did not benefit from laparoscopic intraoperative ultrasound, one patient (4%) had a single functional/surgical kidney, compared with two (3.3%) cases in the ultrasound-guided group.

Patients in the IOUS-guided group were statistically significantly older than those in the non-IOUS-guided group (64 vs. 60 years) and exhibited lower BMIs (Table 1). No gender differences were observed between the two groups, and in both groups, women more frequently underwent either type of surgery (Table 1).

Patients with larger tumors were more likely to be selected for IOUS-guided laparoscopic partial nephrectomy, as confirmed by the significantly higher average tumor size in the IOSU-guided group (3.4 vs. 2.6).

In terms of tumor characteristics, no statistically significant difference was seen in patients regarding the polarity of the tumor, renal pelvis invasion, or urinary tract involvement (Table 1). Most of the cases presented with inferior pole tumors, the renal pelvis was infiltrated in nearly 25% of all cases, and the urinary tract was involved in nearly 40% of cases (Table 1). The majority of tumors in both groups were classified as stage T1b, with no statistically significant difference between groups. Moreover, no difference was seen regarding the histological type of the tumor or the differentiation grade (Table 1).

The laparoscopic approach for performing nephrectomy is particularly important; therefore, the retroperitoneal and transperitoneal techniques were compared, observing statistically significant differences in the duration of the surgical intervention, postoperative food resumption, and average length of hospitalization (Table 2).

Regarding the immediate postoperative follow-up, our study did not show any statistically significant difference in the time until resumption of food, necessary time for postoperative analgesia, and length of hospitalization (Table 3). However, our results proved that patients who underwent IOUS-guided laparoscopic partial nephrectomy suffered less intraoperative bleeding, less ischemia time, and shorter operative time (Table 3). One major intraoperative complication was observed, which consisted of bleeding due to renal pelvis involvement, requiring blood transfusion. Otherwise, no other complication was associated with either of the two techniques.

Using the general linear model, and a Chi-square test, we calculated a power of 0.88, indicating an 88% probability of detecting a true effect. The effect size, estimated post hoc, was 0.353, at a significance level of 0.0012.

## 4. Discussion

In kidney surgery, controversies persist regarding the evolution of technology and the demonstration of its medium and long-term benefits. However, another unquestionable controversy is tied to surgical technique, particularly in terms of approaching the renal tumor. Specifically, there is the possibility of performing laparoscopic partial nephrectomy using either the transperitoneal or retroperitoneal approach, each with its own advantages and disadvantages. Both approaches have demonstrated therapeutic safety [15,22]. Arguments in favor of the transperitoneal approach include a more generous working space, allowing for wider angles and greater maneuverability with laparoscopic instruments, as well as a more familiar orientation based on known anatomical landmarks [23,24]. However, this approach requires mobilization of the intestines to expose the kidney. Intra-abdominal adhesions, which may result from laparoscopic surgeries, seem to have minor clinical significance [15]. On the other hand, the retroperitoneal approach, by avoiding intestinal mobilization, provides more direct access to the kidney and renal hilum. Disadvantages of this method include the spatial limitations of the narrow retroperitoneal working space, the lack of clear visibility, and the risk of disorientation and accidental injuries [25,26]. It is important to note that certain tumors can be successfully approached either through the transperitoneal or retroperitoneal route, depending on the surgeon’s preferences [27].

The retroperitoneal approach provides direct access to the perinephric space, without the need for bowel mobilization. The procedure of retroperitoneal laparoscopic partial nephrectomy has been associated with significant advantages, such as reduced operative time, lower estimated blood loss, and shorter hospitalization compared to the results for the transperitoneal variant of this technique [28]. Another important aspect is the use of continuous two-layer suturing for renorrhaphy. This method has proven to be safe and contributes to a significant reduction in ischemia time and the risk of postoperative bleeding. Additionally, to further minimize ischemia time, the use of Hem-o-lok clips on either side of the suture was added. The result was the absence of any obvious signs of postoperative bleeding, validating the effectiveness of our technique [29,30].

In our center, the decision regarding the laparoscopic approach was left to the discretion of the surgeon and was mainly determined by the location and technical complexity of the tumor mass. The transperitoneal approach was generally preferred for lesions located anteriorly, while the retroperitoneal approach was generally preferred for lesions located posteriorly [15]. The transperitoneal approach was favored for larger tumors or those in hard-to-reach locations [23]. By providing faster access to the kidney and renal hilum, it can be observed that the retroperitoneal approach achieved a shorter average operating time of 117.7 min compared to the transperitoneal approach of 135.4 min, due to the fact that it requires more time for colon mobilization, prolonged dissection of the renal pedicle and the kidney. In their study, Ryuichi Taue et al. demonstrated that there were no significant differences between the two laparoscopic approaches regarding the operating time and blood loss [22]. Still, in our study, a shorter operating time and less blood loss were demonstrated in the retroperitoneal approach compared to the transperitoneal approach. Skills required for the transperitoneal approach are quickly acquired, and the reduction in operating time using the retroperitoneal approach indicates the existence of a learning curve for this procedure [15,31,32].

In hospitalized patients, it is particularly important to assess risk factors for both infections and other pathologies due to increased severity [33]. Thus, increasing body mass index (BMI) is attracting growing attention, especially in developed and developing countries, due to the rising incidence of obesity among the population [34]. Obesity is a real problem and often presents significant challenges for surgeons, simply because it necessitates the use of special equipment and accessories and in many cases, different surgical approaches and increased operative times [35,36,37]. Beyond this aspect, obesity is associated with an escalation of health risks, including conditions such as cardiovascular diseases, diabetes, and respiratory complications. Obesity alone is an independent risk factor for deep vein thrombosis (DVT) development [38]. In our practice, we opt for the exclusive use of pneumatic compression stockings as a prophylactic measure, as recommended by the American College of Chest Physicians at the Pulmonary Congress. Increased attention could be given to adding low-molecular-weight heparin for this high-risk population. A patient with a BMI > 30 requires a longer operating time due to the increased dissection period and difficulty in instrument manipulation. Eliecer Kurzer et al. concluded that laparoscopic surgery presents a level of safety, but each unit increase in BMI increases the risk of experiencing a major complication by 14% [39].

In combination with preoperative computerized tomography (CT) and preoperative ultrasound, IOUS can provide additional real-time information for surgeons, facilitating the assessment of tumor size, number, location, peritumoral vascularization, connection to the renal pedicle, and other tumor characteristics in specific cases [40]. IOUS also offers higher image resolution than does standard transcutaneous abdominal ultrasound and CT, as the IOUS probe is placed directly on the visceral surface during surgery, avoiding interferences caused by abdominal layers and providing a clearer image, allowing surgeons to better focus on details during the procedure [17,41]. Thus, Bhosale et al. present results from a study involving approximately 200 patients, demonstrating a significant alteration in surgical management through the use of intraoperative ultrasound. This is attributed to the observation, during ultrasound, of some changes different from those presented in preoperative imaging investigations, providing new details that require a different approach to the renal tumor compared to that of the initially established plan [42]. Another study, conducted by Polascik et al., yields results that further strengthen our statistical analysis and additionally emphasize the exceptional utility of ultrasound in defining preoperative indeterminate renal lesions [43].

Vascular control, especially the clamping of the renal artery, plays an important role in the success of partial nephrectomy. However, in some cases, there may be accessory renal arteries that cannot be accurately identified by preoperative CT, and their locations cannot be clearly established after dissection during surgery. In this context, IOUS becomes essential for vascular control in laparoscopic partial nephrectomy, helping to shorten the operative time and obtain a high-resolution anatomical image [2,13,14]. Furthermore, with the help of intraoperative ultrasound, a shorter ischemia time, reduced operative time, and lower estimated blood losses were observed. Das et al. also conducted a comparison between conventional laparoscopy and laparoscopy guided by intraoperative ultrasound in a study involving over 100 patients. The results showed clear benefits from using ultrasound guidance, especially in terms of reducing the duration of the surgical procedure. However, the study did not identify statistically significant changes regarding blood loss [44]. The shorter operating time in patients undergoing laparoscopy with ultrasound is primarily attributed to the surgical team’s ability to rapidly identify the tumor and its resection margins. This facilitated a much easier and quicker resection compared to that of conventional methods. In the traditional approach, the margins require additional scrutiny to precisely ascertain whether the resection is adequate or if further resection is warranted. Therefore, surgeons frequently choose to excise larger areas to ensure the complete removal of the tumor mass and minimize the risk of any residual tumor tissue remaining.

Intraoperative complications are more frequent when the renal sinus is involved, with a higher risk of both intraoperative and postoperative bleeding, requiring blood transfusions. There are also other effective methods of suturing, with modern techniques such as the self-retaining barbed suture being notable examples. Studies have demonstrated that this approach can reduce the time required for renal repair and even shorten hospitalization duration. However, it is important to note that these studies have not shown statistically significant results regarding blood loss and operative time [45].

Qin et al. observed results similar to those seen in the patient group examined in this study. However, they also considered additional criteria, leading to particularly important findings. Among these, open conversion and the changes in the glomerular filtration rate for the operated patients (one month post-surgery), where no statistically significant differences were observed between classic laparoscopic surgery and intraoperative ultrasound-guided procedures, are worth noting. The results regarding the glomerular filtration rate indicate that although ultrasound provides a more precise delineation of margins, the additional healthy tissue resected without using intraoperative ultrasound is not substantial enough to significantly disturb the renal balance. However, the patients were evaluated only at one month post-surgery, and we lack conclusive data over a follow-up period of several years, which could indicate the importance of the resected tissue, especially in regards to the overall decline of filtration rate due to reasons other than surgical effects [17].

Another significant advantage of intraoperative ultrasound arises, especially in patients with renal pathology where CT with contrast agents cannot be used. Therefore, during intraoperative procedures, contrast-enhanced ultrasound can be used due to the respiratory elimination of the contrast substance. This approach preserves kidney function and enhances image accuracy [46,47].

Studies by authors such as Senel et al. have explored the potential of using intraoperative ultrasound for exophytic kidney tumors, as demonstrated in a study involving a large cohort of over 500 patients. They attained impressive outcomes, clearly demonstrating reduced ischemia and resection times when intraoperative ultrasound was used [48].

## 5. Conclusions

The use of 3D laparoscopic partial nephrectomy for renal tumors using intraoperative ultrasound prevails as a safe and efficient surgical approach, especially for challenging cases involving completely endophytic tumors located within the renal parenchyma. With the assistance of IOUS, experienced urologists can achieve negative surgical margins, thus allowing for the preservation of healthy renal parenchyma. Furthermore, in patients undergoing surgery with ultrasound guidance, these patients showed lower blood loss, shorter periods of ischemia, and shorter operative duration compared to those undergoing traditional laparoscopic intervention.

## 6. Future Perspectives

Future perspectives are attributed to the ongoing evolution of medical equipment, with a particular focus on robotic surgery. Although robotic surgery is becoming more prevalent, laparoscopy still remains predominant, particularly due to its significantly lower costs compared to robotic surgical procedures. New studies present the advantages of robotic surgery, and successful attempts have been made to combine intraoperative ultrasound (in some cases, even with a contrast substance) with this relatively new type of surgery. The benefits are mainly manifested in higher surgical precision, shorter operative times, and faster discharge of patients from hospital units [49,50,51]. However, further studies are still necessary to conclusively demonstrate the superiority of robotic surgery in terms of the long-term outcomes of patients undergoing partial nephrectomy. Also from a technological advancement perspective, it is crucial to understand that ultrasound has certain limitations. Consequently, several modern and promising systems have emerged, including the three-dimensional augmented reality robot, with the goal of enhancing the management of highly complex tumors [52].

## Figures and Tables

**Table 1:** General characteristics of the patients.

Variable	IOUS-Guided (*n* = 60)	Non-IOUS-Guided (*n* = 25)	*p* Value
*Demographic charateristics*			
**Age, years**	64 (58–68)	60 (54–66)	***0.036***
**Gender**			**0.809**
**Male**	22 (36.3%)	10 (40%)	
**Female**	38 (63.3%)	15 (60%)	
**BMI, kg/m^2^,**	29.5 (25–32)	30 (24–33)	***0.032***
*Tumor characteristics*			
**Solitary Kidney**	2 (3.3%)	1 (4%)	0.449
**Tumor size, cm**	3.4 (2.3–4.2)	2.6 (1.8–3.9)	***0.031***
**Tumor polarity,**			
**Superior**	21 (35%)	6 (24%)	0.444
**Middle**	13 (21.66%)	8 (32%)	0.408
**Inferior**	26 (43.33%)	11 (44%)	0.998
**Clinical TNM stage**			
**1a**	23 (38.33%)	9 (36%)	0.997
**1b**	37 (61.66%)	16 (64%)	
**Endophytic tumor**	40 (66.6%)	15 (60%)	0.622
**Exophytic tumor**	20 (33.3%)	10 (40%)	
**Histology**			
**Clear cell**	56 (93.33%)	22 (88%)	0.441
**Non-clear cell**	4 (6.66%)	3 (12%)	
**Fuhrman grade**			
**G1**	9 (15%)	3 (12%)	0.986
**G2**	40 (66.66%)	18 (72%)	0.799
**G3**	11 (18.33%)	4 (16%)	0.991
**Renal sinus involvement**			
**Not involved**	44 (73.33%)	19 (76%)	0.986
**Involved**	16 (22.66%)	6 (24%)	
**Urinary tract involvement**			
**Not involved**	39 (60%)	17 (68%)	0.977
**Involved**	21 (40%)	8 (32%)	

Categorical variables were presented as count (%) and compared using Fisher’s exact test; continuous variables were presented as median (IQR) and compared using a Mann–Whitney U test.

**Table 2:** The characteristics of different types of approaches and postoperative monitoring.

	Retroperitoneal (*n* = 25)	Transperitoneal (*n* = 60)	*p* Value
Duration of the intervention (min)	117.7 (101–133)	135.4 (112–154)	<0.001
Blood loss (average mL)	132.5 (120–144)	158.8 (139–165)	<0.001
Resumption of food postoperatively (h)	7.4 (4–8.5)	8.9 (7.1–9.8)	<0.001
Postoperative complications	1	0	0.845

**Table 3:** Perioperative complications and early postoperative follow-up.

Variable	IOUS-Guided Group	Non-IOUS-Guided Group	*p* Value
Resumption of food postoperatively, h	8.6 (8.2–8.8)	8.6 (8.2–8.9)	0.072
Postoperative analgesia, h	3.2 (2.8–3.6)	3.4 (2.9–3.6)	0.085
Length of hospitalization, days	5.7 (5.1–6.2)	5.8 (5.2–6.2)	0.092
Postoperative complications	0	1 (4%)	0.294
Blood loss, mL	135 (101–156)	215 (152–258)	<0.001
Operative time, min	115 (98–130)	134 (105–159)	<0.001
Ischemia time, min	19.1 (16.2–23.1)	23.2 (20.2–26.4)	<0.001

## Data Availability

The datasets generated and analyzed during the current study are not publicly available due to institutional restrictions, but are available from the corresponding author upon reasonable request.
